# Development and validation of AI-Enhanced auscultation for valvular heart disease screening through a multi-centre study

**DOI:** 10.1038/s44325-026-00103-y

**Published:** 2026-02-10

**Authors:** Andrew McDonald, Mark Gales, Bushra S. Rana, Matthew Shun-Shin, Benito F. Lukban, Rita Adrego, Alexandros Papachristidis, Fatima Hajee, Len Shapiro, Joanna Wilson, Tony Prothero, Andrew Kennedy, Saul Myerson, Bernard Prendergast, Patrik Bachtiger, Mihir A. Kelshiker, Nicholas Peters, Richard Steeds, Anurag Agarwal

**Affiliations:** 1 Department of Engineering, University of Cambridge, Cambridge, UK; 2 Imperial College NHS Foundation Trust, London, UK; 3 King’s College NHS Foundation Trust, London, UK; 4 Royal Papworth NHS Foundation Trust, Cambridge, UK; 5 National Institute for Health Research (NIHR) Oxford Biomedical Research Centre, Oxford University Hospitals NHS Foundation Trust, Oxford, UK; 6 St Thomas’ Hospital and Cleveland Clinic London, London, UK; 7 University Hospitals Birmingham NHS Foundation Trust, Birmingham, UK

**Keywords:** Valvular disease, Cardiology

## Abstract

Valvular heart disease (VHD) is a growing public health concern, yet over half of cases remain undiagnosed due to late symptom onset, limited public awareness, and low sensitivity of traditional stethoscope-based screening. Current AI-enabled tools rely on murmur detection as a proxy for VHD but lack sensitivity for common subtypes like mitral regurgitation and are limited by small datasets. This study presents a novel neural network that directly predicts clinically significant VHD from stethoscope recordings, trained using echocardiographic targets rather than heart murmur labels. A diverse dataset of 1767 patients across UK primary care and hospital settings was developed, combining stethoscope recordings with echocardiographic labels. The trained recurrent neural network achieved an AUROC of 0.83, outperforming general practitioners and demonstrating exceptional sensitivity for severe aortic stenosis (98%) and severe mitral regurgitation (94%). This algorithm shows promise as a scalable, low-cost screening tool, enabling earlier diagnosis and timely referral for intervention. This research was registered with ClinicalTrials.gov (CAIS: NCT04445012 registered on 2020-06-21, DUO-EF: NCT04601415 registered on 2020-10-19).

## Introduction

Valvular heart disease (VHD) has been described as the ‘next cardiac epidemic’^1^. It is one of the most common causes of heart failure, and the prevalence of degenerative (non-rheumatic) VHD is rising rapidly in developed countries due to an ageing population^2,3^.

More than half of VHD cases remain undiagnosed^2^. Common symptoms, such as breathlessness and fatigue, are often mistaken for respiratory conditions^4^ or attributed to natural ageing, deconditioning or weight gain. Consequently, many patients present late, with advanced symptoms and complications such as heart chamber dilatation, impaired ventricular function, and pulmonary hypertension, resulting in poorer outcomes from interventions. For instance, patients with severe primary mitral regurgitation (MR) treated only after experiencing breathlessness face more than double the risk of heart failure following successful surgery^5^.

Screening for VHD could promote timely intervention, improving patient outcomes and reducing the costly burden of heart failure. Yet, current diagnostic tools are unsuitable for widespread screening. Detection in primary care relies on general practitioners (GPs) using a stethoscope to identify pathological heart murmurs. However, their sensitivity is as low as 45%^6^ and confidence in clinical skills is declining, further contributing to under-use^7^. While echocardiography remains the gold standard for diagnosis of VHD^8^, it currently requires relatively expensive equipment, a detailed examination to be performed with the patient undressed to the waist and is carried out by highly skilled operators who are in limited supply^9^.

An electronic stethoscope device, combined with a machine learning algorithm to interpret heart sounds, could offer an accurate, quick, and accessible screening test for VHD that efficiently identifies patients who need echocardiography^10^. Previous studies have applied murmur detection algorithms that are trained to predict the result of cardiologist auscultation to detect VHD^11–13^. However, these algorithms are unlikely to outperform a human expert and will not learn to detect acoustic features that are inaudible to the human ear or do not fit established patterns, thereby potentially limiting their effectiveness. A further limitation in existing research is the scarcity of large, high-quality datasets: phonocardiograms are not routinely collected in clinical settings, unlike other tests such as echocardiograms or ECGs. Moreover, currently available open-access datasets lack echocardiographic confirmation and are not tailored to adult-acquired VHD^14–16^.

This study aims to design a novel AI algorithm that uses electronic stethoscope recordings to detect clinically significant degenerative VHD. Our objectives are to (i) gather a large dataset from patients with and without various forms of degenerative VHD, (ii) train a new machine learning algorithm using echocardiography as the reference standard, and (iii) evaluate the algorithm’s performance in comparison with that of GPs using a separate test set.

## Results

### Data characteristics

A dataset of 1767 patients (48% female) was collected through three UK NHS studies. The Cardiovascular Acoustics and an Intelligent Stethoscope (CAIS) study was conducted through four acute hospital trusts (Royal Papworth, Imperial, Queen Elizabeth Birmingham, King’s College), and the DUO-EF study^17^ was limited to Imperial College NHS Trust. Additionally, to increase the representation of healthy and asymptomatic patients with milder forms of VHD (who might not typically undergo echocardiography), patients were recruited from primary care settings in collaboration with the OxVALVE population study^2^.

The median age was 74 years (IQR, 66–80 years) and 1,400 (79%) patients were aged 65 or older. A total of 703 (40%) patients were overweight (25 kgm^−^^2^ < BMI < 30 kgm^−2^), and 456 (26%) were obese (BMI > 30 kgm^−2^) (Table 1).

**Table 1:** Baseline characteristics of study patients

Variable		Control (*n* = 974)	Significant VHD (*n* = 793)	Total (*n* = 1767)
Recruitment	Acute hospital	312 (32%)	692 (87%)	1004 (57%)
	Primary care	662 (68%)	101 (13%)	763 (43%)
Sex	Female	468 (48%)	384 (48%)	852 (48%)
	Male	506 (52%)	409 (52%)	915 (52%)
Age (years)	<45	57 (6%)	62 (8%)	119 (7%)
	45%–65	120 (12%)	157 (20%)	277 (16%)
	65%–80	644 (66%)	350 (44%)	994 (56%)
	80+	153 (16%)	224 (28%)	377 (21%)
BMI (kgm^−2^)	Underweight ( < 18.5)	8 (1%)	19 (2%)	27 (2%)
	Healthy (18–25)	275 (28%)	287 (36%)	562 (32%)
	Overweight (25–30)	419 (43%)	282 (36%)	701 (40%)
	Obese (30 + )	265 (27%)	191 (24%)	456 (26%)
NYHA	I	300 (31%)	251 (32%)	551 (31%)
	II	92 (9%)	200 (25%)	292 (17%)
	III	18 (2%)	74 (9%)	92 (5%)
Heart rhythm	Atrial fibrillation	28 (3%)	154 (19%)	182 (10%)
	Other	9 (1%)	29 (4%)	38 (2%)
	Pacemaker	12 (1%)	24 (3%)	36 (2%)
	Sinus rhythm	920 (94%)	577 (73%)	1497 (85%)

Categorical variables are reported as frequency (proportion).

Every patient received a detailed auscultation with a CE-marked electronic stethoscope, and a gold-standard echocardiographic examination performed by a qualified physiologist. Example heart sound recordings are provided in Supplementary Fig. 1. Heart sound recordings yielded 6,479 recordings across four standard auscultation locations (collectively comprising 25 hours of heart sound audio), with 1758 (99%) patients having recordings at the aortic, tricuspid, and mitral sites.

Using a stratified minimisation algorithm, 263 new patients were assigned to the test set and 973 to the training set. Combined with previously collected OxVALVE data, this made a full training set of 1,504 patients. A full flow of study participants is provided in Supplementary Fig. 2.

### Primary outcome

The primary outcome of the study was to identify patients with clinically significant VHD, defined following the OxVALVE study as the presence of equal to or more than mild stenosis or moderate regurgitation in one or more valves^18^. This definition excludes cases of mild regurgitation, which are expected in a large proportion of the study population and do not generally warrant follow-up investigation^2^. This binary classification was chosen to support the device’s potential use as a VHD screening tool, aligning with a ‘refer’ or ‘do not refer’ pathway

Clinically significant VHD, detected with echocardiography was observed in 793 patients (45%) (Table 2). The most common significant valve lesion was aortic stenosis (AS, *n* = 325), followed by MR (*n* = 287). Many patients presented with multiple valve diseases; 258 patients (15%) had more than one significant valve lesion, and 1066 (60%) had mild regurgitation affecting one or more valves. Atrial fibrillation was present in 182 (10%) of patients.

**Table 2:** Distribution of valvular heart disease by severity

	Identified patients, frequency (proportion)								
	Any	AS	AR	MS	MR	PS	PR	TS	TR
Severe	341 (19.3%)	173 (9.8%)	29 (1.6%)	10 (0.6%)	75 (4.2%)	0 (0.0%)	1 (0.1%)	0 (0.0%)	85 (4.8%)
Moderate	409 (23.1%)	91 (5.1%)	157 (8.9%)	11 (0.6%)	212 (12.0%)	1 (0.1%)	32 (1.8%)	0 (0.0%)	194 (11.0%)
Mild	603 (34.1%)	61 (3.5%)	384 (21.7%)	19 (1.1%)	530 (30.0%)	2 (0.1%)	284 (16.1%)	0 (0.0%)	476 (26.9%)
Trace/None	414 (23.4%)	1442 (81.6%)	1197 (67.7%)	1727 (97.7%)	950 (53.8%)	1764.0 (99.8%)	1450 (82.1%)	1767.0 (100%)	1012 (57.3%)

*NB* multivalve disease was common, and patients may be counted more than once in the table. *AS* aortic stenosis, *AR* aortic regurgitation, *MS* mitral stenosis, *MR* mitral regurgitation, *PS* pulmonary stenosis, *PR* pulmonary regurgitation, *TS* tricuspid stenosis, *TR* tricuspid regurgitation.

### Algorithm performance

**Fig. 1:**
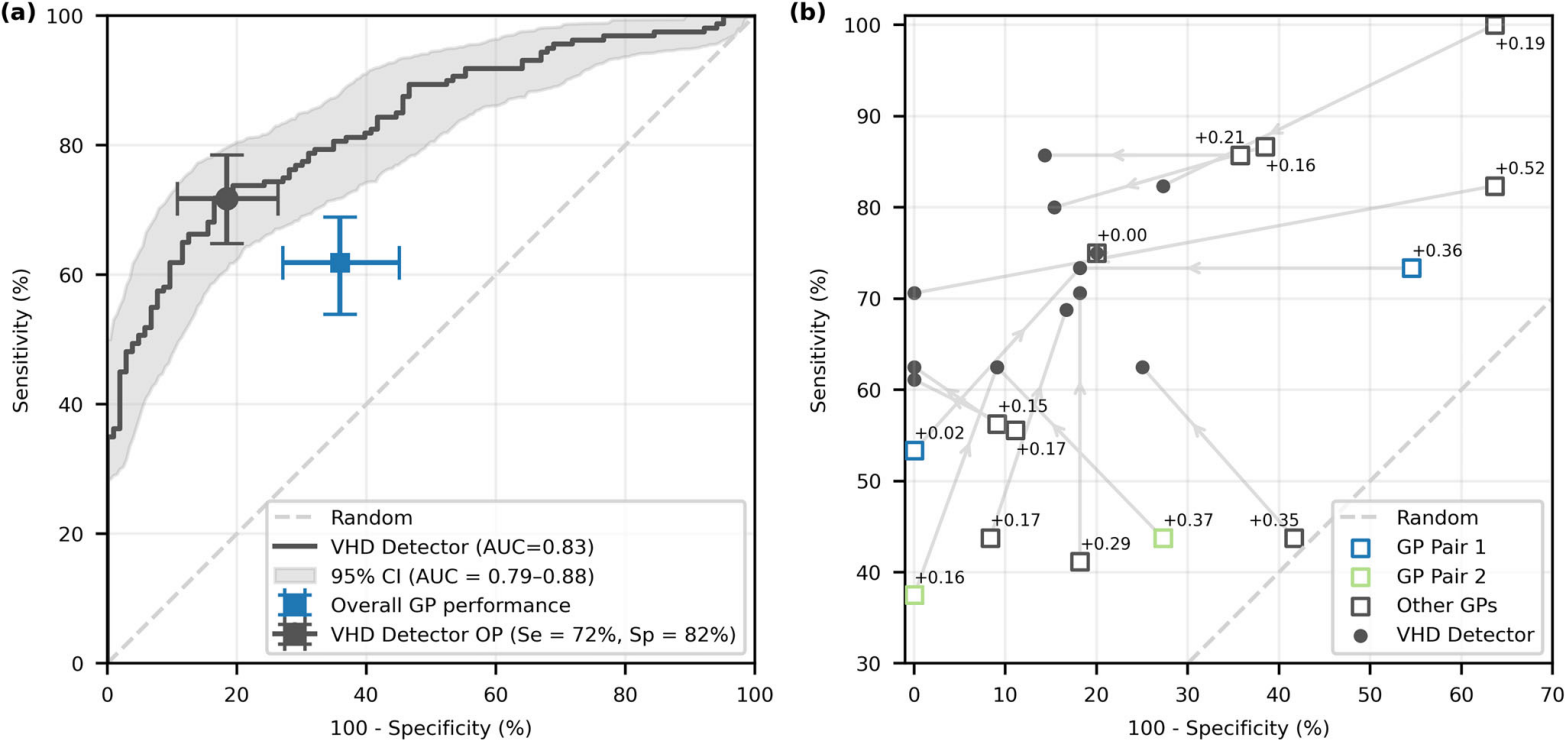
Receiver operating characteristic curves demonstrating the comparison of algorithm and GP performance on the test set. **a** Overall performance of the algorithm and GPs, with 95% confidence intervals generated through iterative bootstrapping. **b** Comparison of individual GP performance. Arrows link the performance of each GP to the corresponding performance of the algorithm on the same patient set. Two pairs of GPs saw the same patients, as highlighted. The change in Youden index between the GP and the algorithm is shown next to each GP performance.

A recurrent neural network algorithm, trained using with echocardiographic labels as the reference standard (the ‘VHD Detector’), achieved an AUROC of 0.83 (95% CI: 0.79–0.88) for identifying clinically significant VHD (Fig. 1a). At the designated operating point (threshold probability ≥ 0.675), the algorithm demonstrated a sensitivity of 72% (95% CI: 65–79%) and a specificity of 82% (95% CI: 74–89%). This operating point was chosen retrospectively as a potentially suitable threshold for a screening device. A full confusion matrix for this result is provided in Supplementary Table 1.

The VHD Detector’s predicted probabilities also demonstrates strong calibration with the echocardiographic gold standard, with an expected calibration error of 0.08 (95% CI: 0.06–0.11). Calibration is critical for clinical application, as it ensures that the predicted probabilities can be reliably interpreted to make informed decisions. For instance, a well-calibrated algorithm provides confidence that a prediction of a 70% likelihood of significant VHD corresponds closely to a true 70% chance of the condition being present (see reliability diagram, Supplementary Fig. 3).

The cross-validated training and test set performances of the VHD Detector are closely aligned (Supplementary Fig. 4), particularly in the high specificity region, providing confidence that the model did not significantly overfit to the training data. As the test set is small compared to the training set (*n* = 263 vs. *n* = 1504), we report additional metrics on the training set for reference.

### Performance across valve types and severities

We note that the severity mix within the positive class influences the ROC results in Fig. 1a, and a breakdown of the sensitivity of the VHD Detector across various significant VHD types operating at the previously established probability threshold (with a fixed specificity of 82%) is provided in Table 3. Notably, on the test set the VHD Detector shows a 98% sensitivity for severe AS (95% CI: 90–100%) and 94% sensitivity to severe MR (95% CI: 76–100%). Individual ROC curves for significant AS and MR are provided in Supplementary Fig. 5. Test sensitivity is lower for other severe valve lesions that may produce more subtle auscultation findings (for example, tricuspid regurgitation, 83% [95% CI: 61–94%]). Sensitivity for moderate AS remains high at 89% (95% CI: 68–100%), though it is lower for moderate MR, reaching 75% on the test set but only 50% on the cross-validated training set. Moderate MR was also commonly encountered in combination with other significant valve lesions (consistent with clinical practice), and this may have increased sensitivity – particularly in the test set that includes a larger proportion of patients recruited in the hospital setting. Data concerning the sensitivity of the VHD Detector in patients with only one significant valve lesion is provided in Table 3. A total of 17 patients in the test set had isolated moderate MR, and sensitivity in this setting reduced (as expected) to 65%. However, sensitivity remained high for isolated severe AS (100%), moderate AS (100%), and severe MR (88%), though the sample size was reduced in this analysis.

**Table 3:** Diagnostic sensitivity of the VHD Detector across individual severities of clinically significant VHD in both the test and cross-validated training set

		Test		Cross-validated training	
Disease	Grade	Sensitivity	*n*	Sensitivity	*n*
AS	Severe	98% (90–100%)	41	98% (94–99%)	132
	Moderate	89% (68–100%)	19	85% (75–92%)	72
	Mild	80% (50–100%)	10	67% (53–78%)	51
MR	Severe	94% (76–100%)	17	90% (79–97%)	58
	Moderate	75% (58–86%)	36	50% (43–57%)	176
TR	Severe	83% (61–94%)	18	61% (49–72%)	67
	Moderate	53% (36–69%)	36	53% (45–61%)	158
AR	Severe	45% (18–73%)	11	67% (44–83%)	18
	Moderate	58% (38–77%)	26	63% (55–71%)	131
PR	Moderate	50% (17–83%)	6	58% (38–73%)	26
MS	Severe	75% (10–100%)	4	50% (17–83%)	6
	Moderate	100% (n/a)	3	62% (25–88%)	8
	Mild	67% (0–100%)	3	75% (50–94%)	16
Isolated AS	Severe	100% (n/a)	25	97% (91–100%)	67
	Moderate	100% (n/a)	11	85% (73–94%)	48
	Mild	100% (n/a)	3	68% (51–81%)	37
Isolated MR	Severe	88% (38–100%)	8	90% (77–97%)	30
	Moderate	65% (35–82%)	17	41% (31–52%)	85
Isolated TR	Severe	71% (29–100%)	7	50% (33–64%)	36
	Moderate	38% (19–62%)	16	35% (24–47%)	68
Isolated AR	Severe	33% (0–83%)	6	67% (33–92%)	12
	Moderate	12% (0–62%)	8	40% (26–57%)	35

NB: Sensitivity reported as the sample sensitivity (95% CI). Additionally reported is the sensitivity for isolated VHD where only one type of significant VHD is present. The algorithm operates with a fixed specificity of 82% across all combinations. The sample size for moderate MS is too low for accurate calculation of confidence intervals. *AS* aortic stenosis, *MR* mitral regurgitation, *TR* tricuspid regurgitation, *AR* aortic regurgitation, *PR* pulmonary regurgitation, *MS* mitral stenosis.

### Auscultation site analysis

Analysis of auscultation position importance revealed that the tricuspid site was the most important single location, achieving 75% sensitivity for significant AS and 64% sensitivity for significant MR (Table 4). However, incorporating the aortic and mitral sites substantially increased sensitivity across the spectrum of VHD, while adding the pulmonary site had minimal impact on overall sensitivity.

**Table 4:** Variation in VHD Detector diagnostic sensitivity based on different combinations of auscultation sites for four key types of significant VHD in the test dataset

Locations	Sensitivity for significant VHD, value (95% CI)			
	AS (*n* = 69)	MR (*n* = 50)	TR (*n* = 53)	AR (*n* = 37)
AMPT	93% (86–97%)	84% (72–92%)	62% (48–75%)	54% (38–70%)
AMT	93% (86–97%)	80% (66–90%)	60% (47–72%)	51% (35–68%)
MT	81% (71–88%)	74% (60–84%)	55% (42–68%)	41% (27–57%)
AT	91% (84–97%)	72% (58–82%)	55% (42–68%)	49% (32–65%)
A	72% (61–81%)	42% (28–55%)	25% (15–40%)	41% (27–57%)
P	68% (57–78%)	60% (46–74%)	40% (26–53%)	30% (16–46%)
T	75% (64–86%)	64% (50–76%)	49% (36–62%)	35% (19–51%)
M	57% (45–67%)	58% (46–70%)	36% (25–51%)	30% (16–46%)

Across all used locations, the maximum probability was used to predict significant VHD. The algorithm operates with a fixed specificity of 82% across all combinations. *A* aortic, *P* pulmonary, *T* tricuspid, *M* mitral, *AS* aortic stenosis, *MR* mitral regurgitation, *TR* tricuspid regurgitation, *AR* aortic regurgitation.

### Comparison with general practitioners

To contextualise algorithm performance, 14 UK GPs with varying clinical experience (0-28 years) were recruited to make predictions on the test dataset using electronic stethoscope recordings (see Supplementary Table 2). The test set was divided into 12 subsamples, with six patients included in all subsamples to assess inter-observer agreement.

The overall performance of the GPs on the test set is also shown in Fig. 1a, where the final GP prediction assigned to an individual patient is a majority rule from all GPs (with a bias to refer if there is a tie). The ensembled GP performance achieves a sensitivity of 62% (95% CI: 55–70%) and specificity of 64% (95% CI: 54–73%). The VHD Detector significantly outperforms GP predictions with respect to both sensitivity (*p* = 0.01) and specificity (*p* = 0.002), highlighting its potential as a reliable screening tool. Contingency tables for this McNemar test are provided in Supplementary Table 3.

The performance of each GP compared to the algorithm is illustrated in Fig. 1b. The operating points of individual GPs varied widely, with some prioritising sensitivity and others focusing on specificity. In contrast, the algorithm’s performance was more consistent, clustering in a high-specificity region. Notably, the algorithm outperformed each GP, achieving a higher Youden Index in 13/14 cases and matching GP performance in the remaining case. This result underscores the algorithm’s superior balance of sensitivity and specificity across the test set.

In addition, paired GPs, who predicted on the same patient data, produced very different results. For example, GP Pair 1 only agreed on 17 of 26 patients, with one GP classifying 9 extra patients as clinically significant VHD compared to the other. Amongst the 6 shared patients assessed by all GPs, there was general agreement on 3, including 2 with severe AS. However, there was disagreement on patients with mild MR, trace VHD, and severe aortic regurgitation. Further breakdown on GP inter-observer agreement is provided as Supplementary Information.

## Discussion

This multi-centre study demonstrates that a machine learning algorithm can effectively detect clinically significant VHD using heart sound recordings at standard chest auscultation sites. Combined with an electronic stethoscope, the algorithm has the potential to serve as a quick, non-invasive screening tool for moderate or severe VHD, offering improved sensitivity and consistency than current primary care clinical algorithms, and triaged access to currently limited (and expensive) echocardiographic services.

The amalgamated dataset in this study constitutes one of the world’s largest and most comprehensively labelled phonocardiogram resources. The availability of a paired echocardiographic gold standard for every patient enabled detailed analysis of algorithm results on a wide range of acquired VHDs of varying severity. With data collected from multiple clinical settings and by clinicians of varying experience levels, the dataset reflects a realistic usage environment, thereby enhancing the model’s generalisability.

A key strength of the VHD Detector is its high sensitivity for significant AS and severe MR, conditions with distinct acoustic signatures that are common and of high clinical importance. This high sensitivity translates to a high negative predictive value in a screening environment, providing GPs with greater confidence to exclude severe left-sided VHD. However, sensitivity was lower for other valve lesions with more subtle and complex acoustic features. Nevertheless, our accuracy in detecting significant VHD exceeds those of previously described murmur detection algorithms, even though these studies often excluded poor-quality signals, which can artificially inflate performance metrics. For example, Roquemen-Echeverri et al.^19^ rejected 21% of recorded signals, achieving a sensitivity of 88.9% and 63.3% for moderate-severe or greater AS and MR, respectively. Similarly, Chorba et al.^12^ reported a sensitivity of 93% for moderate-to-severe or greater AS and 66% for moderate-to-severe or greater MR, whilst Waaler et al.^13^ showed a high sensitivity of 98.7% for moderate or greater AS but only 56.3% for moderate or greater MR. They noted that the main limitation of their approach was that their algorithms would miss important acoustic features beyond murmur grade, thereby limiting the detection of VHD—a limitation also identified by Roquemen-Echeverri et al.^19^ While direct comparisons are challenging due to differences in datasets, our approach reflects real-world conditions by including all recordings regardless of signal quality.

A notable distinction of our work is that the VHD Detector algorithm was trained to detect clinically significant VHD directly from echocardiographic labels rather than via murmur detection^11–13^. This approach leads to a model with strong calibration and discrimination, potentially allowing the future development of continuous risk scores to aid clinical decision-making. In contrast, murmur detection studies have not reported calibration performance^12,13^, which is likely to be inferior since significant VHD without an audible murmur would be confidently rejected. Moreover, direct echocardiographic training offers advantages by avoiding the complexity and substantial interobserver variability of murmur annotations^11^. The dataset also consists of data from two types of digital stethoscope, giving confidence that the VHD Detector algorithm could be integrated into existing off-the-shelf devices for easier deployment.

We note that the algorithm exhibited decreased sensitivity for moderate forms of VHD, particularly for regurgitant valve diseases. This was expected and is likely due to the murmurs from moderate regurgitation being quieter and harder to detect using a stethoscope alone. Whilst not as a immediately clinically significant as severe disease, effective detection of moderate cases is important for patient monitoring and planning timely intervention. Expanding the number of examples of these diseases in future work would improve the algorithm’s ability to localise these quieter sounds and improve overall sensitivity.

Although our dataset included diverse manifestations of VHD, it was not representative of a UK screening population. To provide sufficient VHD cases for model training and evaluation, we intentionally biased recruitment from hospital sites (including valve clinics). Real-life screening populations would have a lower prevalence of symptomatic and severely diseased patients, resulting in both a lower overall sensitivity and a lower positive predictive value. For this key reason, we prioritised the specificity of the test, following existing screening test examples^20^. Future prospective studies should model the effect of lower disease prevalence and incorporate health economic analysis to determine the overall impact of population screening. We also note that our study focused on degenerative instead of congenital or rheumatic VHD, which are also important to detect but will manifest in a younger population with different acoustic characteristics.

The size of the withheld test set, though smaller than ideal, still provided a reliable measure of performance, since the cross-validated training set performance closely aligned with the test set performance. This alignment indicates that the model’s performance should generalise to larger datasets. However, sub-analyses for some specific valve lesions (e.g., mitral stenosis) or grades of severity levels were limited by the small sample sizes.

Comparisons with the GPs performance provided encouraging evidence that the algorithm improves the accuracy and consistency of a clinical assessment. Nevertheless, we acknowledge that a significant limitation of this comparison is that the study GPs did not have direct access to patients and so did not perform an in-person auscultation. Listening via headphones may have impacted their ability to detect subtle murmurs and the quality and tonality of sound from an electronic stethoscope differs from a standard one. This limitation may constrain the wider generalisability of this result. A larger, prospective study would be valuable to evaluate GP performance in real-world conditions.

In conclusion, the VHD Detector shows promise as a screening tool for acquired VHD, enabling timely referral of patients with moderate and severe VHD that warrant echocardiography and further follow-up, whilst excluding those with mild regurgitation or trace VHD that requires no further action. Unlike more complex screening methods such as handheld echocardiography, this tool requires only one minute of simple stethoscope recordings, making it quick, accessible, and easily deployable by unskilled operators with minimal training. Future prospective pilot studies in primary care settings will be essential to validate performance in real-world environments, generate trust among healthcare providers, and enable earlier detection of VHD.

## Methods

### Study design

Adult patients (aged > 16 years) in hospital-based studies were included if they were attending for routine transthoracic echocardiography as part of their clinical assessment. Patients were excluded if they had previously undergone valve replacement or repair, were pregnant, or were in New York Heart Association (NYHA) Class IV heart failure^21^ (indicating severe symptoms at rest). The OxVALVE study included patients aged > 65 years with no previous diagnosis of VHD^22^. All three studies provided patients with and without VHD.

All participants provided written informed consent, and all constituent studies were approved by the UK Health Research Authority (CAIS: 15/YH/0541, DUO-EF: 21/LO/0051, OxVALVE: 09/H0502/58). The hospital-based studies were registered with ClinicalTrials.gov (CAIS: NCT04445012 registered on 2020-06-21, DUO-EF: NCT04601415 registered on 2020-10-19). This study adheres to the STARD reporting guidelines^23^ and a checklist is included as Supplementary Table 4.

### Clinical examination

All patients underwent auscultation with a CE-marked electronic stethoscope (either the 3M Littmann Model 3200 or Eko DUO), performed by a cardiologist, cardiac physiologist, or research nurse. Patients were asked to sit upright with their chest exposed, breathing normally, and any bra was loosened or removed if applicable. Heart sounds were recorded for up to 15 s at four standard auscultation locations: aortic, pulmonary, tricuspid, and mitral sites. Examinations took place in typical clinical settings, such as outpatient rooms, with associated ambient noise. Unlike previous studies, we did not apply signal quality screening to the recordings, thereby providing a more realistic indication of algorithm performance under real-world conditions. Clinicians who recorded heart sounds were not blinded to the patient’s clinical results.

Transthoracic echocardiography, performed by a qualified physiologist, was used as the gold standard for VHD diagnosis. The severity of identified VHD was locally graded at each site as trace, mild, moderate, or severe, according to pre-specified British Society of Echocardiography guidelines^24^. To ensure consistency in reporting across sites, a CoreLab reviewed a sample of echocardiograms from each recruitment site. This review found that echocardiographic labels were, in general, highly consistent across sites. While most echocardiograms (94%) were conducted on the same day as the auscultation, a window of up to three months was allowed, provided there were no substantial changes in the patient’s clinical condition. Echocardiographers were not blinded to the patient’s auscultation results or other clinical findings.

### Statistical analysis

Categorical variables describing the cohort are reported using proportions and frequency. Non-normal continuous variables are reported using the median and interquartile ranges.

An unseen test set was used to evaluate the algorithm’s generalisation to new data. Throughout the study, newly recruited patients were prospectively allocated to either a training or withheld test set using a stratified minimisation algorithm that balanced key prognostic variables such as recruitment site, type, and grade of VHD. The test set was withheld from the AI research team until the end of the study. Patients from the OxVALVE cohort who had been recruited before the main study were automatically allocated to the training set.

Sensitivity and specificity comparisons between the AI algorithm and GPs were conducted using the McNemar test for two correlated proportions^25^ and a significance level of 5%. GP inter-observer agreement was assessed using Cohen’s Kappa and Fleiss’ Kappa analysis. Calibration was assessed through reliability diagrams and expected calibration errors^26^ and iterative bootstrap re-sampling used to generate 95% confidence intervals.

### Algorithm development

All analyses were performed in Python, using the PyTorch deep learning library, NumPy, and SciPy. The design of the AI algorithm was finalised retrospectively after study completion, but before access to the test set was granted.

The study’s machine learning model builds on a murmur detection and segmentation algorithm that won the 2022 PhysioNet challenge^27,28^. While traditional approaches focus solely on murmur detection, our model extends this foundation to specifically identify clinically significant VHD, using a recurrent neural network to analyse time-frequency representations of audio recordings and detect murmurs and other acoustic features associated with significant VHD. A recurrent neural network was chosen as the foundational model because of the limited training set available, which made larger-parameter approaches (such as transformers or deep convolutional neural networks) more likely to overfit to training examples in early experiments. Before being input to the network, recordings were denoised using a spike-removal algorithm^29^ before being rescaled to unit amplitude, transformed into a Mel-frequency spectrogram, and z-normalised to increase the energy in higher frequencies and normalise the transfer function of the different stethoscopes^27^.

A transfer learning approach was applied, starting with open-access data from the PhysioNet 2016 and 2022 challenge datasets to pre-train the model for murmur detection^14,16^. The parameters from this initial model were then refined on the new data collected in this study using echocardiographic labels for significant VHD, resulting in the final ‘VHD Detector’ algorithm. Model hyper-parameters, such as the hidden layer size and spectrogram, were optimised through five-fold cross-validation on the training set.

Medical training indicates that different types of VHD are best detected at different auscultation sites^30^. For instance, murmurs of MR are often most audible at the apex, while those related to AS murmurs radiate from the aortic site through the tricuspid site. We therefore trained an independent machine-learning model for each site, allowing each model to learn the expected sounds. The maximum probability across all locations was taken to generate an overall prediction for each patient since detection of VHD should only require an abnormal sound from a single valve.

### Clinician comparison

Given that recruitment was predominantly from hospital referral clinics, it was not feasible for GPs to examine the patient with their own stethoscope. Instead, each GP received an online survey containing electronic stethoscope recordings for each assigned patient. To ensure audio quality, GPs were instructed to use high-quality headphones and confirm that they could clearly hear a sample recording. They were then asked to predict whether each patient had clinically significant VHD. The GPs were blinded to all clinical variables except for patient sex.

## Supplementary information

Supplementary Information

## Data Availability

The datasets generated and/or analysed during the current study are not publicly available due to clinical ethics requirements but are available from the corresponding author on reasonable request for research use. Interested parties should contact the corresponding author. Any data sharing will be subject to meeting data protection rules, and may require institutional review board approval as appropriate.

## Code availability

The underlying code for this study is not publicly available but may be made available to qualified researchers on reasonable request from the corresponding author.
