# Supplementary material for: Development and validation of AI-Enhanced auscultation for valvular heart disease screening through a multi-centre study
Source: NPJ Cardiovasc Health. 2026 Feb 10;3:5. doi: 10.1038/s44325-026-00103-y (PMC12890582; doi:10.1038/s44325-026-00103-y)
Supplement: Supplementary file 1 — Supplementary Information [file 44325_2026_103_MOESM1_ESM.pdf]

Development and Validation of AI-Enhanced Auscultation for Valvular Heart Disease Screening through a Multi-Centre Study

Supplementary Information

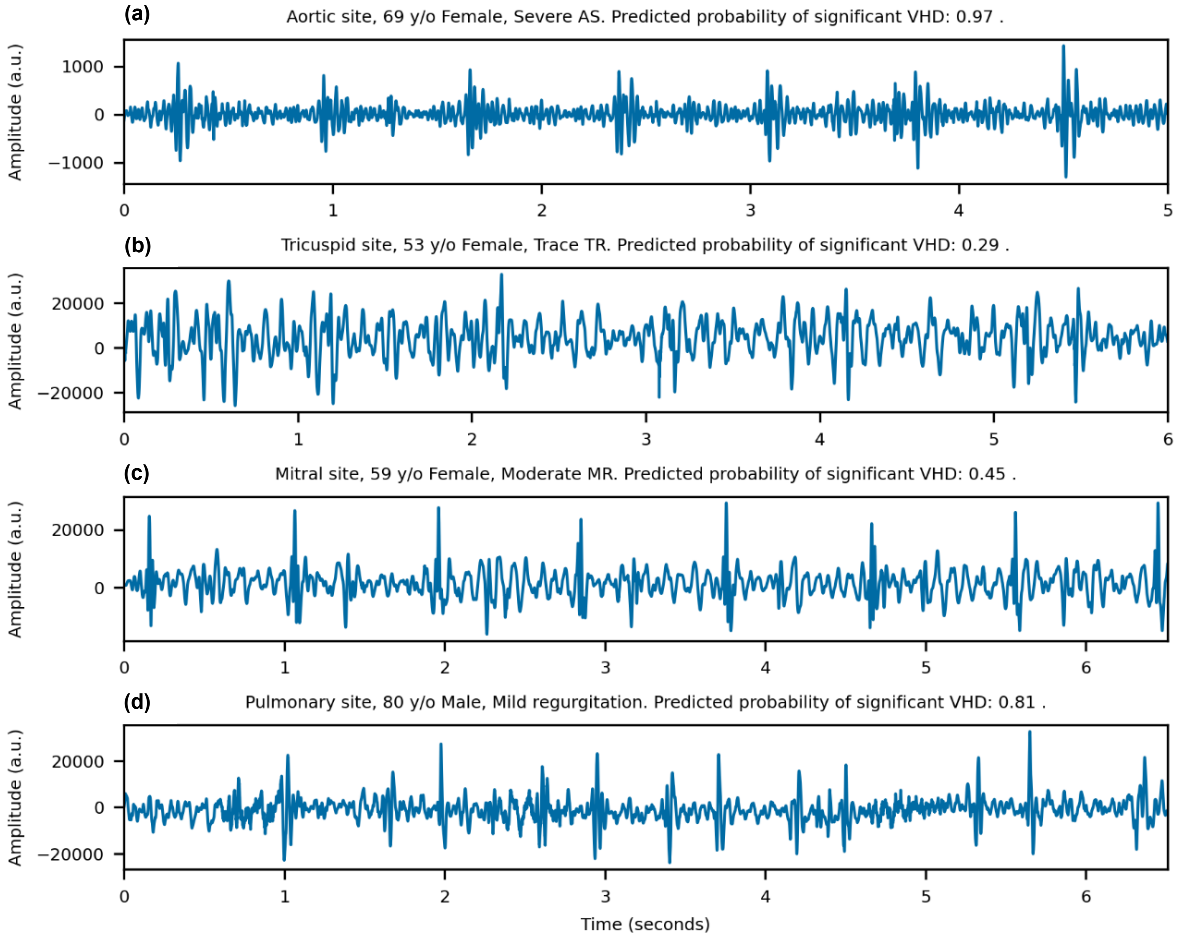

Supplementary Figure 1. Sample electronic stethoscope heart sound recordings. (a) true positive, (b) true negative, (c) false negative, and (d) false positive. Sample (a) was recorded using the Eko DUO stethoscope, whereas the others were recorded using the Littmann 3200 stethoscope.

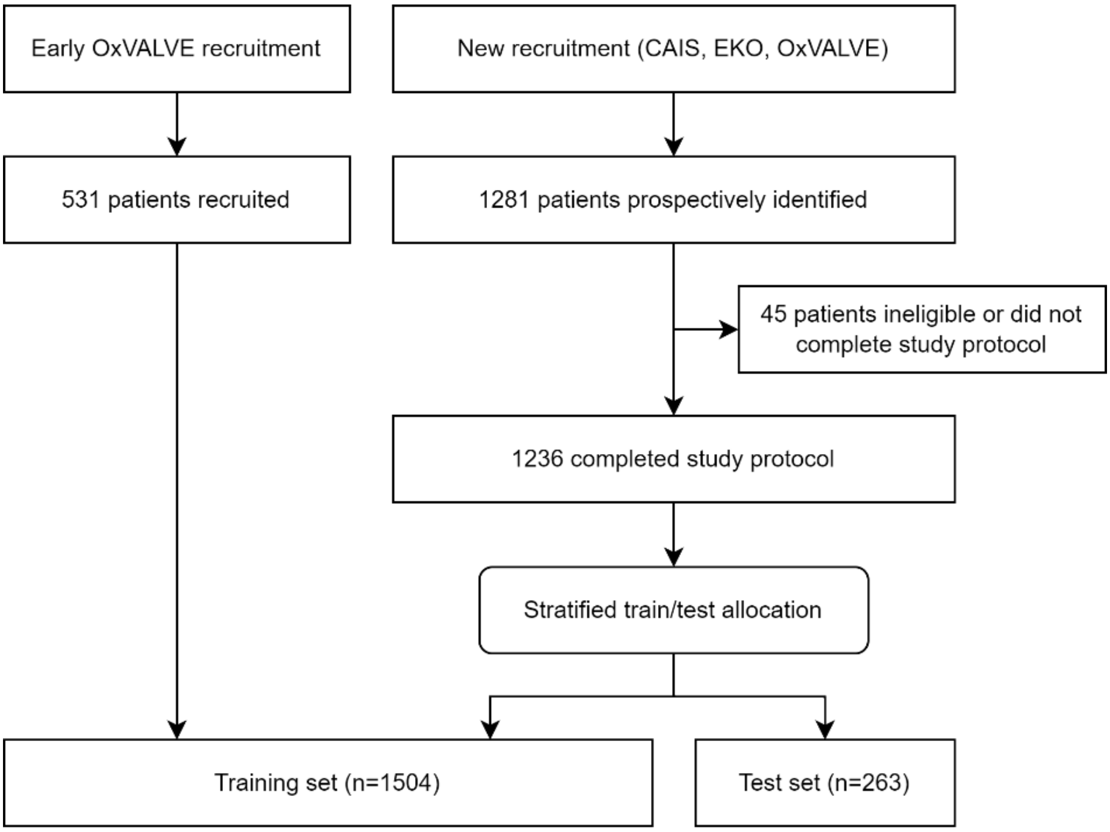

Supplementary Figure 2. Flow of study participants. A total of 1767 patients were included in the study, split between a training set of 1504 patients and a test set of 263 patients.

Supplementary Table 1. Confusion matrix for algorithm prediction on test set, with gold-standard split by maximum severity of VHD in an individual patient.

	No or Insignificant VHD			Significant VHD		
	None	Trace	Mild regurgitation	Mild stenosis	Moderate	Severe
Prediction negative	7	21	56	1	32	12
Prediction positive	1	3	15	3	41	71

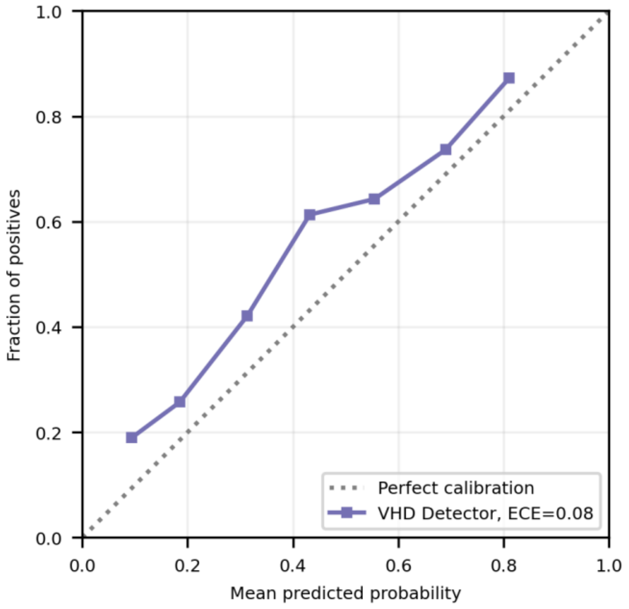

Supplementary Figure 3. Calibration performance of VHD Detector at predicting clinically significant VHD, compared to gold standard echocardiography. The figure shows a reliability diagram that illustrates the calibration of the algorithm and with its expected calibration error (ECE). An algorithm that produces perfectly calibrated probabilities would lie on the diagonal line.

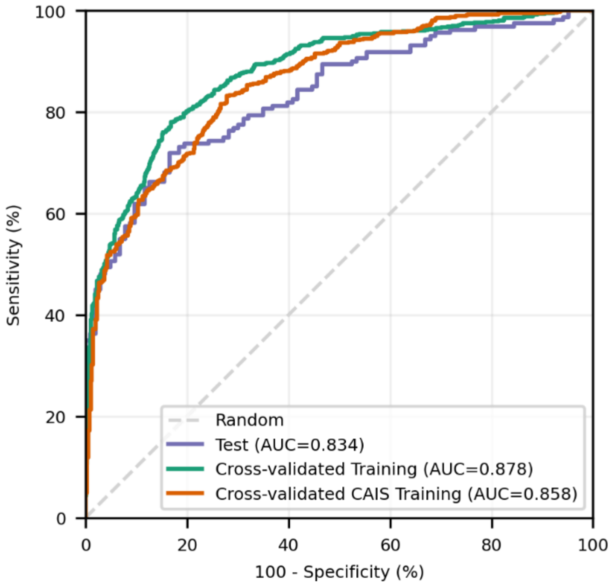

Supplementary Figure 4. Receiver operating characteristic curve illustrating performance of the VHD Detector on different datasets. Shown here is the performance of the algorithm on the test set, the cross-validated training set, and a subset of the cross-validated training set with the same disease distribution as the test set.

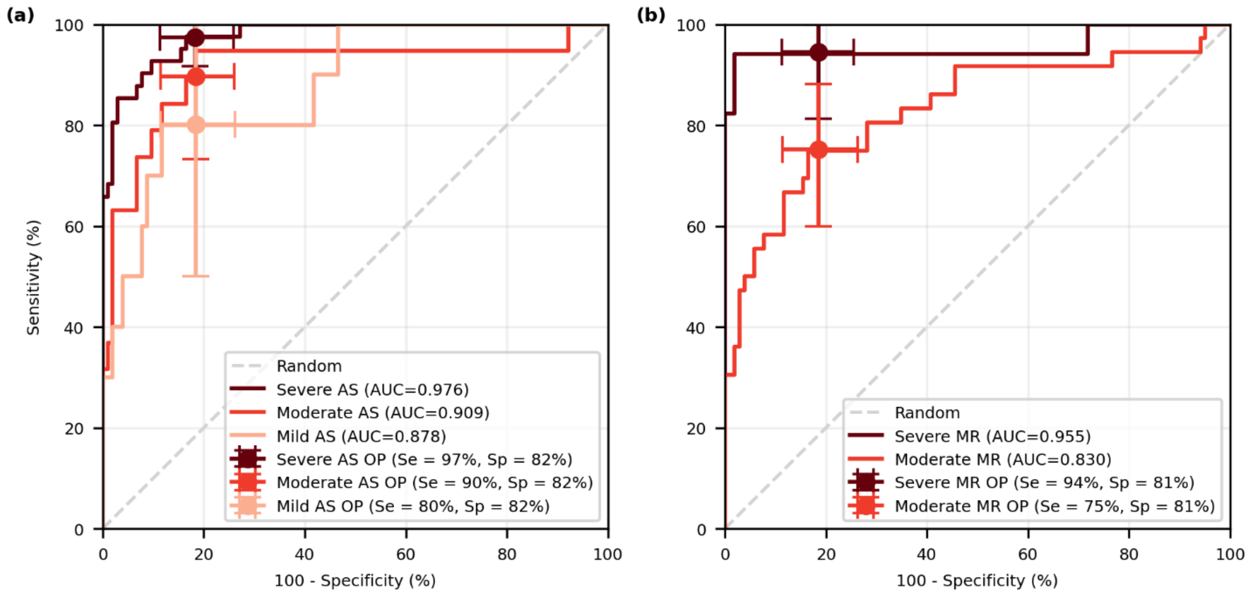

Supplementary Figure 5. Receiver operating characteristic plots for algorithm detection of clinically significant (a) aortic stenosis and (b) mitral regurgitation in the test set. For each plot, an ROC curve was plotted for the detection of individual grades of disease compared to control patients with no (or insignificant) VHD. Mild mitral regurgitation was considered an insignificant condition and included as a control case.

Supplementary Table 2. Experience levels of general practitioners who took part in the study survey. 14 GPs were recruited to listen to one of 12 subsamples of the test set and 2 pairs of GPs (4/5 and 13/14) therefore listened to the same patient set.

Sample ID	GP ID	Years qualified in current role	Previous cardiology experience		
			Medical school	Medical training	Speciality roles as GP
1	1	12	Yes	Yes	No
2	2	25	Yes	Yes	Yes
3	3	24	Yes	No	Yes
4	4	1	Yes	No	No
	5	0	Yes	Yes	No
5	6	5	Yes	Yes	No
6	7	28	Yes	Yes	No
7	8	27	Yes	Yes	No
8	9	6	Yes	Yes	No
9	10	6	Yes	No	No
10	11	1	Yes	No	No
11	12	1	Yes	No	No
12	13	1	Yes	No	No
	14	25	Yes	Yes	No

Supplementary calculations of GP inter-observer agreement.

On the six patients seen by all 14 GPs:

		Number of GPs (out of 14)	
**Patient**	**VHD**	**Predicted negative**	**Predicted positive**
1	Mild regurg.	10	4
2	Severe AS	0	14
3	Severe AS	0	14
4	Healthy/Trace	10	4
5	Mild regurg.	14	0
6	Severe AR, Moderate PR	7	7

The resulting Fleiss Kappa score was 0.527, indicating moderate agreement.

Sample 4 (GP 4 & 5):

		GP 5 Prediction	
		Negative	Positive
GP 4 Prediction	Negative	9	9
	Positive	0	8

The corresponding Cohen's Kappa score was 0.381, indicating only fair agreement.

Sample 12 (GP 13 & 14):

		GP 14 Prediction	
		Negative	Positive
GP 13 Prediction	Negative	16	1
	Positive	5	5

The corresponding Cohen's Kappa score was 0.481, indicating moderate agreement.

Supplementary Table 3. Contingency tables for McNemar comparison of GP and algorithm predictions on the test set. Negative and positive patients in the test set (n=263) were considered separately, to allow statistical comparison of specificity and sensitivity, respectively.

	No/Insignificant VHD		Significant VHD	
	Algorithm negative	Algorithm positive	Algorithm negative	Algorithm positive
GP negative	59	7	35	26
GP positive	25	12	10	89

Supplementary Table 4. STARD 2015 checklist for reporting diagnostic accuracy studies. Signposts are provided to demonstrate how each STARD item was addressed in this study.

Section and Topic	No	Item	Reported in section
**Title or abstract**			
	1	Identification as a study of diagnostic accuracy using at least one measure of accuracy (such as sensitivity, specificity, predictive values, or AUC)	Abstract
**Abstract**			
	2	Structured summary of study design, methods, results, and conclusions (for specific guidance, see STARD for Abstracts)	Abstract
**Introduction**			
	3	Scientific and clinical background, including the intended use and clinical role of the index test	1
	4	Study objectives and hypotheses	
**Methods**			
Study design	5	Whether data collection was planned before the index test and reference standard were performed (prospective study) or after (retrospective study)	2.1
Participants	6	Eligibility criteria	2.1
	7	On what basis potentially eligible participants were identified (such as symptoms, results from previous tests, inclusion in registry)	2.1
	8	Where and when potentially eligible participants were identified (setting, location, and dates)	2.1
	9	Whether participants formed a consecutive, random, or convenience series	2.1
Test methods	10a	Index test, in sufficient detail to allow replication	2.2
	10b	Reference standard, in sufficient detail to allow replication	2.2
	11	Rationale for choosing the reference standard (if alternatives exist)	1
	12a	Definition of and rationale for test positivity cut-offs or result categories of the index test, distinguishing pre-specified from exploratory	3.2
	12b	Definition of and rationale for test positivity cut-offs or result categories of the reference standard, distinguishing pre-specified from exploratory	2.2
	13a	Whether clinical information and reference standard results were available to the performers or readers of the index test	2.2
	13b	Whether clinical information and index test results were available to the assessors of the reference standard	2.2
Analysis	14	Methods for estimating or comparing measures of diagnostic accuracy	2.4
	15	How indeterminate index test or reference standard results were handled	n/a
	16	How missing data on the index test and reference standard were handled	3.1
	17	Any analyses of variability in diagnostic accuracy, distinguishing pre-specified from exploratory	3.2
	18	Intended sample size and how it was determined	n/a
**Results**			
Participants	19	Flow of participants, using a diagram	Figure S1
	20	Baseline demographic and clinical characteristics of participants	Table 1
	21a	Distribution of severity of disease in those with the target condition	Table 2

	21b	Distribution of alternative diagnoses in those without the target condition	
	22	Time interval and any clinical interventions between index test and reference standard	2.1
Test results	23	Cross tabulation of the index test results (or their distribution) by the results of the reference standard	Table S1
	24	Estimates of diagnostic accuracy and their precision (such as 95% confidence intervals)	3.2
	25	Any adverse events from performing the index test or the reference standard	n/a
**Discussion**			
	26	Study limitations, including sources of potential bias, statistical uncertainty, and generalisability	4
	27	Implications for practice, including the intended use and clinical role of the index test	4
**Other information**			
	28	Registration number and name of registry	2.1
	29	Where the full study protocol can be accessed	2.1
	30	Sources of funding and other support; role of funders	2.1
